# Freeze-Cast Chitosan/Resole Aerogels: Effect of Resole Fraction on Properties and Their Efficiency for Cr(VI) Uptake

**DOI:** 10.3390/gels12040330

**Published:** 2026-04-15

**Authors:** Jean Flores-Gómez, Milton Vázquez-Lepe, Álvaro de Jesús Martínez-Gómez, Víctor Hugo Romero-Arellano, Juan Morales Rivera

**Affiliations:** 1 Departamento de Agua y Energía, Centro Universitario de Tonalá, Universidad de Guadalajara, Tonalá 45425, Mexico; 2 Departamento de Ingeniería de Proyectos, Centro Universitario de Ciencias Exactas e Ingenierías, Universidad de Guadalajara, Zapopan 45100, Mexico; milton.vazquez@academicos.udg.mx; 3 Departamento de Ingeniería Química, Centro Universitario de Ciencias Exactas e Ingenierías, Universidad de Guadalajara, Guadalajara 44430, Mexico; alvaro.mgomez@academicos.udg.mx; 4 Departamento de Ciencias Básicas Aplicadas e Ingeniería, Centro Universitario de Tonalá, Universidad de Guadalajara, Tonalá 45425, Mexico; hugo.romero@academicos.udg.mx

**Keywords:** chitosan aerogel, resole phenolic resin, unidirectional freeze-casting, chromium (VI) adsorption, XPS

## Abstract

Aligned CS/Rx aerogels were fabricated by inducing non-directional ice growth (freeze-molding) followed by low-temperature curing, resulting in monoliths with interconnected channels, a high void fraction, and moldability. The swelling index (S%) was calculated to be 1029, the apparent density 0.496 g·cm^−3^, and the estimated porosity 90% based on micrographic analysis. Aerogels have mechanical behavior Shore A hardness greater than 25. Batch metal removal tests were performed (10 mL, 100 mg·L^−1^ Cr(VI), 0.19 g adsorbent, 24 h, and pH 5–5.5), and the material achieved 95% metal removal. Additional kinetic and isothermal results were obtained using CS85R15 on a packed column (20 to 140 mg·L^−1^, 1000 mL Cr(VI), 0.80 g adsorbent, 24 h, and pH 5–5.5). Equilibrium data were consistent with a heterogeneous surface hosting a specific site, as reflected in the joint Freundlich/Langmuir fit (q_max_ 100.8 mg·g^−1^ for Langmuir). This confirmed the preservation of chitosan functionalities (–OH/–NH) after processing, while XPS detected chromium on the surface with signals consistent with the partial reduction of Cr(VI) to Cr(III) on the aerogel surface. This highlights the relevance of adsorption-based technologies for water remediation, where high-porosity and low-density materials allow for short diffusion pathways and capture electrostatics by protonated amines and redox conversion of hazardous substances. The soft-cure freeze-molding technique is simple, scalable, and compatible with packed-bed/column operation, providing a material platform for tailoring the microstructure (sheets and channels) and surface chemistry to regenerable sorbents for industrial wastewater treatment.

## 1. Introduction

The demand for efficient methods to remove contaminants from water continues to grow worldwide, particularly when heavy metal contamination is present. Functionalized materials have emerged as promising tools for this purpose [1,2]. Among them, chitosan (CS) has attracted significant attention due to its amine-rich polysaccharide backbone, which originates from the deacetylation of chitin obtained from fish waste [3,4,5], making it economical and sustainable. Its metal-binding capacity, mediated by electrostatic and chemical interactions of the –NH_2_ groups, has been extensively documented [6,7,8]. However, practical implementation requires controlling swelling and mass transfer limitations to ensure selective and efficient adsorption [6,7,9]. Porous adsorbents with large surface areas generally exhibit better ion removal performance [2,3,10]. In this sense, freezing and melting allow the manufacture of biomimetic materials with aligned sheets and a large specific surface area [11], potentially improving the internal diffusion pathways relevant for adsorption [10,11,12,13,14]. Despite the structural advantages of freeze-molded chitosan, these aerogels often exhibit brittleness and fragility, and the process can involve complex control over ice nucleation and growth orientation, increasing manufacturing costs and reducing scalability [9,15]. To overcome these limitations, incorporating phenol–formaldehyde resole resin into the chitosan suspension represents a promising strategy. Resole resins (R) are synthesized under basic conditions [16,17,18,19] and are characterized by their ability to cure without additional crosslinkers, remaining soluble long enough to be mixed with acidic CS solutions before solidification [10,16,17,18]. Once frozen and cured, the resole forms methylene and ether bridges that reinforce the scaffold, improving mechanical integrity while preserving chitosan as the main functional component for metal adsorption [20]. We propose that controlling the resole fraction (x) allows for the adjustment of key structural parameters (lamellar architecture, porosity, and swelling resistance), which in turn impact Cr(VI) adsorption, as stabilized porous networks improve mass transfer and maintain the accessibility of adsorption sites [21,22]. Although CS-based materials have been extensively studied in the form of films [23,24], fibers [25,26,27], membranes [12,24,28,29], beads [30,31], microspheres [32,33], coatings [34,35] and hydrogels [33,36,37,38], and chitosan aerogels have been explored for contaminant removal, existing studies focus on the addressing how the resole fraction governs structure–property–adsorption relationships in freeze–melt CS/R aerogels remain scarce [10,22]. In this work, a series of CS/R aerogels was fabricated by refrigerator freezing with unidirectional freeze-casting and low-temperature curing. This study analyzes whether, during aerogel molding, there is a resole fraction that maximizes the balance between chitosan-NH_2_ accessibility, aligned lamellar porosity, and mechanical integrity, thereby improving Cr(VI) uptake. The main objective was to quantify the effect of the resole fraction on (i) microstructure (SEM) [10], (ii) swelling index and porosity [19,39], (iii) mechanical hardness/compression) [40], and (iv) Cr(VI) removal capacity [8,39].

## 2. Results and Discussion

### 2.1. CS-x Aerogel Characterization

**Figure 1:**
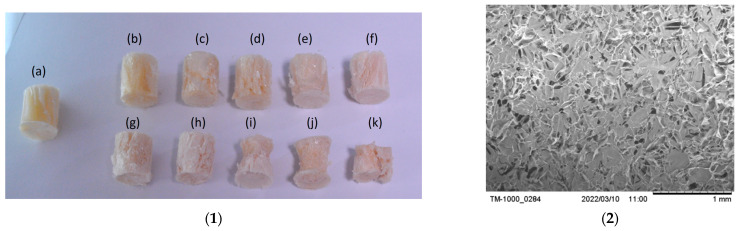
(**1**) Final geometry structure obtained from the cylindrical recipient of CS-x aerogels without thermal treatment (**a**) CS100/R0, (**b**) CS95/R5, (**c**) CS90/10, (**d**) CS85/R15, (**e**) CS80/R20, (**f**) CS75/R25, (**g**) CS70/R30, (**h**) CS65/R35, (**i**) CS60/R40 (**j**) CS55/R45 and (**k**) CS50/R50; (**2**) SEM micrograph magnification of (**a**) CS100/R0 without resole.

**Figure 2:**
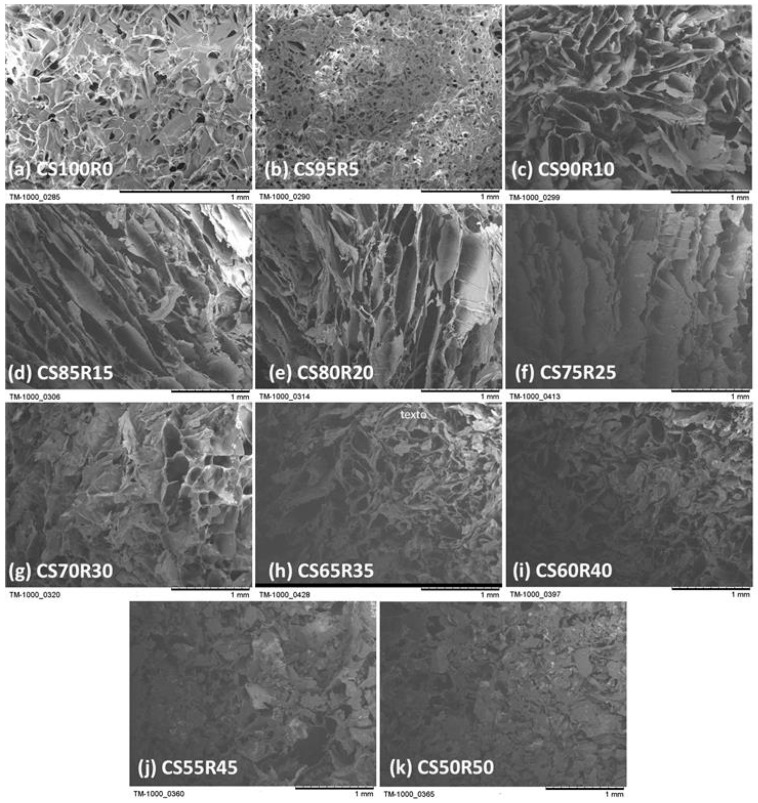
Cross-section SEM micrographs for different resole ratio formulations, lamellar internal structure decreases and porous structures are observed from CS100/R0 to CS50/R50 proportional resole ratios from (**a**–**k**).

Figure 1(1a) shows CS/R x aerogels after demolding and before thermal curing; the monoliths are flexible and exhibit no measurable hardness, with a marked decrease in size after demolding. After curing, the yellowing and loss of flexibility are consistent with resole curing and aging, which typically increase mechanical integrity in freeze-molded networks, as described by Maleki et al. [9]. In Figure 1(1b) (CS100/R0), dilute chitosan molds a porous scaffold by freeze-molding, producing a dense, high-area network; 100× micrographs show a uniform distribution of nanometer-sized pores [10,41,42]. The porosity values obtained from an SEM image analysis represent apparent two-dimensional porosity, which is suitable for comparative morphological evaluation. All the CS/R x aerogels exhibited high porosity (>85%), consistent with freeze-molded aerogel architectures. Increasing the chitosan fraction led to a progressive decrease in porosity, accompanied by a marked reduction in pore size and an increase in sheet thickness, indicating structural densification and partial collapse of the ice-molded network [43]. These results demonstrate that porosity alone is insufficient to describe transport accessibility, and that pore size and sheet thickness must be considered to identify an optimal composition window for aerogel formulation [44]. A change is observed with increasing CS fraction and lower resole, as shown in Figure 2a–c. Morphologically, it is quite notable that the pore diameter decreases abruptly (92 to 97% vs. the composition with the highest resole loading) and the lamellar thickness increases, with a simultaneous drop in porosity (47% at the end). This pattern suggests solid-phase compaction and loss of lamellar order, reducing the effective connectivity of the channels [43]. Conversely, the formulations with the highest resole loading (R 50–75, Figure 2g–k exhibit large pores (median 600–1850 µm) and thin lamellars (2.6–7.8 µm) while maintaining high porosity (92%). This lamellar architecture with open channels favors percolation and seemingly straight or linear trajectories, facilitating water delivery and internal diffusion to active adsorption sites [44]. A decrease in pore size accompanied by an increase in sheet thickness is consistent with structural densification and partial ice-tempered network collapse, as Karimi et al. suggest [45]. Therefore, we can infer that the formulations of CS/R aerogels in an apparently better ratio, as shown in Figure 2d–f, are most suitable. Thus, pore size and sheet thickness should be considered together to identify an optimal composition window for aerogel formulation as reported [46]. The incorporation of resole into the formulation aims to reinforce and stabilize the structure generated during ice growth, regardless of slight variations in the freezing front’s trajectory. This behavior can also be attributed to thermodynamic considerations, as the gel–solvent system tends to minimize its free energy during solidification, favoring entropy-induced self-assembly processes within the polymer scaffold. It is assumed that the freezing front advances mainly in an axial direction, from the meniscus towards the bottom of the tempered cristal tube, traveling a characteristic distance equal to the height of the cylinder at an estimate rate of R ≈ 0.80 cm h^−1^.

### 2.2. Swelling Experiments

The results of the swelling experiments are summarized in Table 1. CS/R-x aerogels with lower resole fractions exhibit a very high swelling index (S%), reaching S% ≈ 5000 for CS100/R0, which is consistent with superabsorbent-like behavior reported for chitosan-based hydrogels under specific crosslinking conditions [16,17,26,47,48]. The decrease in S% with increasing resole content reflects the progressive network densification and reduced hydrophilicity imparted by the phenolic resin. In this framework, the inverse correlation between S% and apparent density is physically reasonable for a higher density and lower S%; therefore, the amount of resole prevents the walls from being flexible [49].

### 2.3. Shore A Hardness Measurements

**Figure 3:**
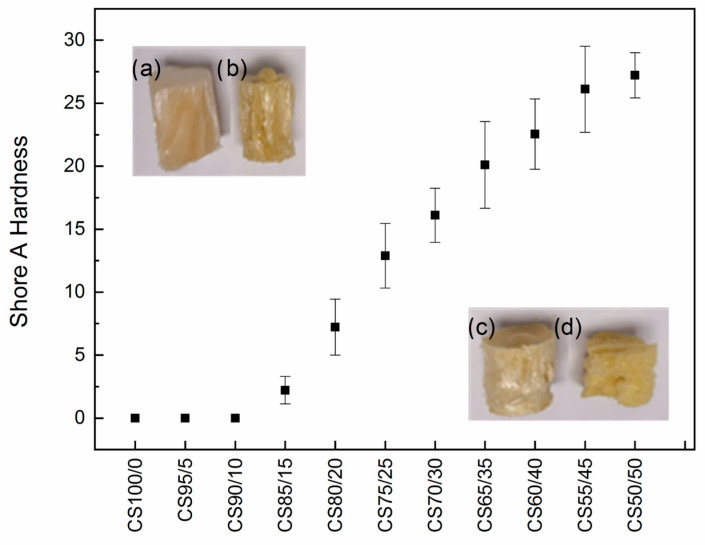
Shore measurement of the hardness of the CS-x aerogels after the thermal treatment. Samples of CS/R x aerogels: (a) CS100/R0, (b) CS85/R15, (c) CS70/R30 and (d) CS50/R50.

Shore A hardness was measured following standard durometry practice (multiple indents per specimen; results as mean ± SD). The resole fraction exerted a crucial effect: CS50/R50 reached Shore A ≳ 25, while the samples with low resole (e.g., CS100/R0, CS95/R05, and CS90/R10) could not be measured because the indenter penetrated completely, indicating soft/elastic behavior. Thermal treatment was necessary to complete the resole curing, enabling reproducible hardness values [50]. The observed hardness trend is consistent with the literature on polymer-crosslinked aerogels, where higher crosslink density leads to higher modulus/hardness while maintaining low density [51,52], as observed in Figure 3.

### 2.4. FT-IR Analysis

**Figure 4:**
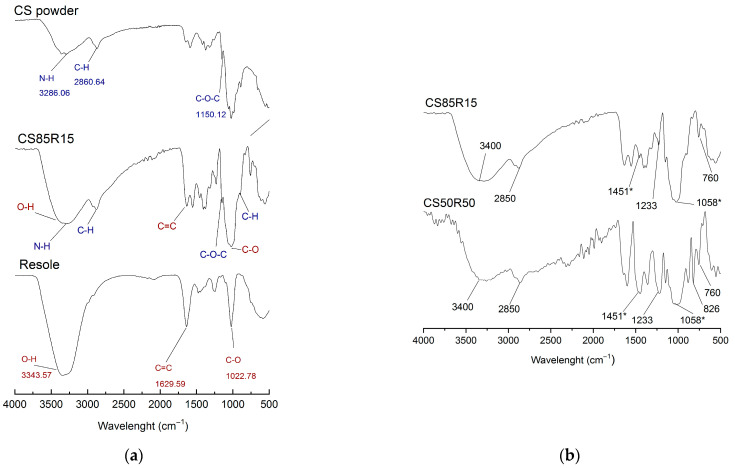
(**a**) FT-IR comparison of chitosan powder, CS85R15 aerogel and resole (Critical functional groups (blue and red) are visible on CS85R15; (**b**) FT-IR comparison of CS85R15 aerogel and CS50R50 aerogel (* intensification of the bands associated with methylene bridges (1450 cm^−1^) and ether bonds (C–O–C 1100 cm^−1^).

Figure 4 compares FT-IR spectra of chitosan, resole precursor, and CS-x aerogels. For chitosan, the broad ν (OH,NH) band appears at 3200–3500 cm^−1^, with Amide I at 1645 cm^−1^, Amide II at 1560–1590 cm^−1^, Amide III at 1320 cm^−1^, ν (C–O–C)/ν (C–O) at 1020–1070 cm^−1^, and a glycosidic signal near 890 cm^−1^ characteristically of saccharide and a crystalline sensitive band on 659 cm^−1^ [26,40,48,49,53]. For the resole, we observe a broad phenolic ν (OH) at 3200–3500 cm^−1^, aromatic C=C at 1600–1500 cm^−1^, methylene (CH_2_) deformation near 1450 cm^−1^, C–O stretching of the –CH_2_OH groups around 1040–1060 cm^−1^, and out-of-plane C–H for para- (830 cm^−1^) and ortho-substituted rings (750–760 cm^−1^) [16,17,47,54,55,56,57]. In CS/R-x aerogels, increasing the resole fraction intensifies bands associated with methylene bridges (1450 cm^−1^) and ether linkages (C–O–C 1100 cm^−1^), consistent with thermal curing/reticulation; consecutively, –CH_2_OH (1050 cm^−1^) tends to decrease as condensation proceeds [40,58]. The broad 3200–3500 cm^−1^ envelope reflects overlapping ν (OH)/ν (NH) with hydrogen-bonding contributions from both networks. Overall, the spectral evolution supports chemical crosslinking between the phenolic resin and the polysaccharide network during curing [25].

### 2.5. XPS Analysis

**Figure 5:**
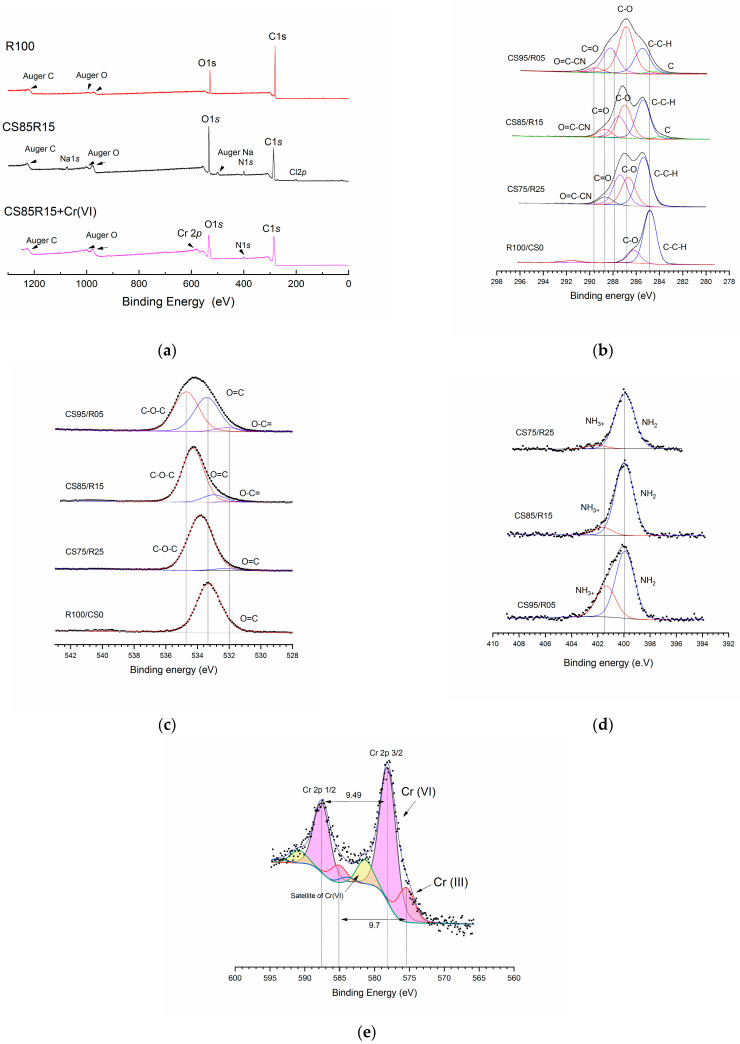
This figure shows the XPS analysis comparisons: (**a**) survey of resole, CS-x aerogel and Cr (VI) loaded CS85/R15; (**b**) carbon 1*s* comparison high-resolution spectra; (**c**) oxygen 1*s* comparison high-resolution spectra; (**d**) nitrogen 1*s* comparison high-resolution spectra; (An increase or decrease in the number of electrons is observed from the corresponding deconvolutions. The features are depicted using the same color, as they correspond to the same bond identified). (**e**) Cr2p high-resolution spectra.

The analysis of XPS spectra (C1s, O1s, N1s, and Cr2p) were acquired from resole (R100/CS0) and CS/R-aerogels (CS75/R25, CS85/R15, and CS95/R05), as well as from CS85/R15 after Cr(VI) adsorption, as observed in Figure 5a. The survey spectra show the presence of C and O signals characteristic of the phenolic network in the resole study; the CS-x spectra show C, O, and N (the latter contributed by chitosan). Cr2p is detected in Cr-laden CS85/R15, confirming chromium capture.

**Figure 6:**
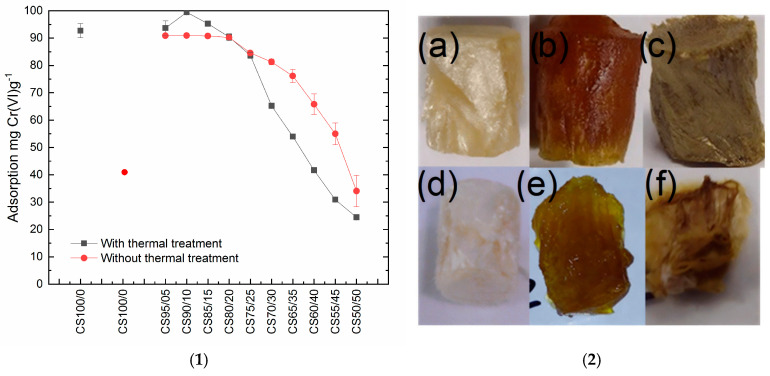
(**1**) Adsorption of batch experiments with and without thermal treatment. (**2**) Pieces of CS-aerogel CS90/10 with thermal treatment, (**a**) dry, (**b**) wet, (**c**) dry with Cr(VI) after adsorption, and CS-aerogel CS90/10 without thermal treatment (**d**) dry, (**e**) wet, (**f**) dry with Cr(VI) after adsorption.

As seen in C1 (Figure 5b, in resole, the main C–C/C–H component was fixed at 284.8 eV; a second component of 286 eV attributable to C–O/C–N was observed, and a third of 288–289 eV associated with C=O/O–C=O. In CS-x, the review requires 4 to 5 components: C–C/C–H (284.8–285.4 eV), C–O/C–N (286.2–286.9 eV), C=O (288–289 eV), and, depending on the formulation, a contribution of N–C=O of 289–290 eV (amide) [29,57]. Increasing chitosan (CS95/R05) increases C–O/C–N (286.8 eV) and decreases C–C/C–H, consistent with a higher polysaccharide fraction, and agitation peaks > 291–292 eV may appear [56], as seen in O1 Figure 5c. Two or three components were resolved: C=O (531.5–532 eV) and C–O/C–O–C (533–535 eV), with a possible minor contribution of 532 eV (amide/hydroxyl). In CS95/R05, C–O/C–O–C broadens (534.7 eV) and C=O remains constant (533.1 eV), which is consistent with a higher chitosan content appearing [56] as seen in N1 (Figure 6(2d). Two components are identified: –NH_2_ (399.8–400.0 eV) and –NH_3_^+^/amide (401.2–401.5 eV). With higher chitosan concentrations, the N content increases, and in acidic (acetic) medium, the protonated fraction (–NH_3_^+^) is favored. So, despite the fact that we have a higher concentration of N1s, the percentage protonated by the acidification of the mixture limits the amino group present [35,59], as seen in Cr2p on CS85/R15 after adsorption (Figure 5e). The main component, Cr2p3/2, at 576.6–577.1 eV, is assigned to Cr(III); a component at 579.5–580.5 eV indicates remnant Cr(VI). It is recommended to adjust only 2π/2 and use multiplets for Cr(III) and one peak for Cr(VI). Consider the reduction of Cr(VI) → Cr(III). This suggests that, during adsorption, the reducing surface with NH_2_ and the hydroxyl group generates the reduction of Cr(VI) through electrostatic attraction. The NIST X-ray database also identifies the peak at 573.5 eV as Cr(III), which is almost entirely reduced on the QS85/R15 aerogel [33,56,57,59,60,61].

From the deconvolution summarized in Table 2, the resole shows a C1s dominated by C–C/C–H at 284.8 eV (68%), with a second component at 286.1 eV (14%) attributable to C–O/C–N; in O1s, 533.0 eV (18%) predominates, consistent with C–O/C–O–C in polymeric matrices, while the signal at 539.9 eV corresponds to loss (“shake-off”/inelastic loss) (the bond energies are referenced to C1s = 284.8 eV). When chitosan is incorporated (CS75/R25 → CS85/R15 → CS95/R05), a systematic decrease in C–C/C–H is observed (31% → 22% → 16% at 285.3–285.4 eV) with an increase in the 286.8–286.9 eV component (21.3% → 23.3% → 29%), indicating a surface enrichment in C–O/C–N characteristic of the polysaccharide; in addition, the C=O components at 288–289 eV are maintained/modulated according to the resole fraction, as expected in phenol–formaldehyde networks. In O1s, the contributions of 534–535 eV (CS75/R25: 26.0% → CS85/R15: 26.2% → CS95/R05: 30.0% adding up to 534.7 + 533.4 + 532.1 eV), characteristic of C–O/C–O–C, increase, while the contributions of 532–533 eV are associated with C=O/amide/hydroxyl, confirming a net increase in oxygenated functionalities with higher chitosan content. In N1s, the total surface fraction increases (CS75/R25: 2.4%; CS85/R15: 6.0%; and CS95/R05: 6.3%) and is dominated by the 401.3 eV component versus 399.9 eV, indicating that most of the nitrogen appears as –NH_3_^+^/amide (high BE) and the remainder as neutral –NH_2_ (low BE), consistent with protonation in an acidic preparation/adsorption medium and with the increase in the chitosan phase. In conclusion, XPS counts reveal that, with increasing chitosan fraction, the CS-x surface becomes enriched in the C–O/C–N and N groups, maintaining the C=O signals associated with the resole. This chemical evolution supports the idea that the amino/hydroxyl functionality of chitosan becomes more exposed/abundant, while the resole provides the underlying aromatic and carbonyl skeleton [35,59].

### 2.6. Batch Adsorption Experiments

This study evaluated whether the different CS/R-x aerogels exhibit adsorption capacity. All the CS/R-x materials adsorbed Cr(VI); however, higher resole fractions reduced the uptake, which we attribute to lower swelling/permeability and a decrease in accessible surface contributed by the chitosan template. To highlight the role of aging/curing, we first tested CS/R-x aerogels without the final thermal treatment, as observed in Figure 6(1) The thermal step chemically crosslinks the polymeric matrix, providing the desired mechanical integrity, but it also limits swelling, which can diminish mass transfer. Consequently, for high-resole formulations, non-cured samples removed more Cr(VI) than their thermally cured counterparts. The effect of aging was particularly evident for CS100/0: thermal treatment strengthened the internal chitosan-derived network; without it, the adsorption decreased, and the monolith disintegrated in acidic Cr(VI) solution, behaving like powdered, gel-forming chitosan. After adsorption, the CS/Rx-aerogel retained its shape on drying and exhibited a metallic coloration consistent with chromium species on the composite surface, as observed in Figure 6(2b).

### 2.7. Column Adsorptions Experiments

**Figure 7:**
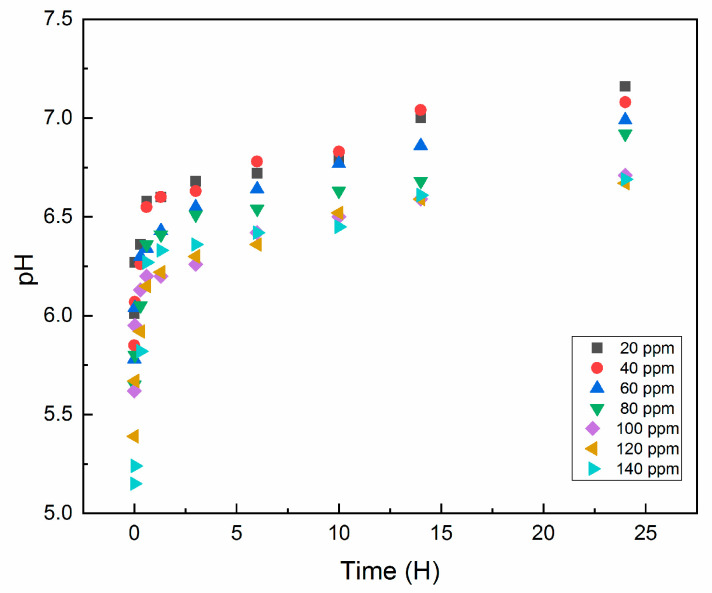
pH evolution during the adsorption kinetics at 24 h from the concentrations of 20 to 140 (mg·L^−1^).

Bibliography indicates that Cr(VI) adsorption with chitosan occurs efficiently in a pH range of 2 to 5 [62]. In this work, the adsorption column design required that the chromium solution not be adjusted or conditioned, since chitosan acquires a positive charge in acidic media, which favors the electrostatic equilibrium between the protonated amino groups (–NH_3_^+^) and the anionic Cr(VI) species [5], hence the importance of pre-washing the adsorbent to restore near-neutral initial conditions, more details in Supplementary Materials. Figure 7 shows the pH evolution during sampling and the adsorption kinetics at 24 h. Initially, the Cr solution has an acidic pH close to 5; however, as the effluent repeatedly passes through the adsorbent material, a progressive decrease in ionic interaction is observed, attributed to the reduction of free species in the solution. This leads to a gradual neutralization of the medium to a pH of approximately 7 at the end of the experiment. Isotherm analysis Table 3. indicates that the experimental data fit the Langmuir and Freundlich models, adjustment of models (Figures S1–S4) in Supplementary Materials. However, the Freundlich model shows a better statistical fit (R^2^ = 0.9786), suggesting a heterogeneous adsorption process with multilayer formation and a predominance of electrostatic physical interactions. Furthermore, the significant fit to the Langmuir model (R^2^ = 0.9634; q_max_ = 100.8 mg·g) demonstrates the simultaneous presence of chemisorption at specific sites, which was subsequently corroborated by chemical surface characterization techniques. Taken together, these results indicate that the adsorption of Cr(VI) onto QS85/R15 occurs through a mixed mechanism, dominated by a heterogeneous surface with contributions from both physisorption and chemisorption.

As observed in Table 4, a comparison with CS-based adsorbents reported in the literature that most chitosan-based Cr(VI) adsorbents achieve high Qmax values under strongly acidic conditions (pH 2–3) and batch static operation, frequently requiring relatively high adsorbent dosages. In contrast, the CS85/R15 work operates at a milder pH (5–5.5) using a low adsorbent mass while maintaining competitive adsorption capacity.

## 3. Conclusions

CS/R-x aerogels effectively integrate structural robustness and adsorbent functionality, offering formation of aligned lamellar structures; anisotropic growth; and the generation of large, interconnected pores, favorable for applications in adsorption and fluid transport, for the retention and transformation of Cr(VI) in batches and columns. The combination of lamellar microstructure, heterogeneous surface, and amino groups activates a mixed mechanism with competitive q_max_ and a process compatible with scale-up. Consolidating their practical application requires further research into the redox mechanism, and optimization, for example, specifically measuring breakthrough curves (C/C_0–t_, dynamic capacity) under different residence times, bed heights, and operating cycles, as well as evaluating regeneration or operating cycles, could be a promising area for future research. In addition, the ecotoxicity and carbon footprint of the process will be assessed. From a techno-economic perspective, CS/R-x aerogels combine low reagent costs, moderate energy consumption during processing, and significantly improved mechanical durability. While freeze-drying accounts for most of the energy consumption, the low-temperature curing (~60 °C) results in a favorable operational balance. Ideally, a life cycle assessment (LCA) and a cost analysis should also be performed. Further investigation would be conducted on the composition window and properties, column design and regeneration, and safety and sustainability assessments. These lines of research will strengthen the technological viability of CS/R-x aerogels in water and waste management and facilitate their industrial adoption.

## 4. Materials and Methods

### 4.1. Matrix Concept and Materials

Chitosan powder > 85% deacetylation degree was obtained from America Alimentos, Zapopan, Mexico; HCl (37.0%), CH_3_COOH (99.9%), C_6_H_5_OH (99%), H2CO (37.0%), NaOH (98%), (CH_3_)_2_CO (99.0%), and H_2_SO_4_ (97.3%) were bought from Fermont (Productos Químicos Monterrey, Monterrey, Mexico). Additionally, deionized water and ethanol were used for dissolved and washing solutions, respectively.

Chitosan was prepared at 2 wt.%, with an acetic acid solution of 2 vol.% without mechanical stirring, dissolving in repose for three days at room temperature. The chitosan solution is the principal agent of the scaffold formation, and it establishes the growth of ice crystals as the main matrix with >50 vol.% of gel. An amount of 20 wt.% water content is suitable for the nucleation of water, which is influenced by this state of gel during freezing [48,66].

Formulation of the aerogel composite process.

### 4.2. Synthesis of Phenol–Formaldehyde Resins for CS-x Blends

Resole solution of phenol–formaldehyde resin was prepared by the reported method [15] with adjusted modifications [22], and the monomer resole precursor was synthesized by adding formaldehyde and phenol into a solution of NaOH (0.1 M). The mixture was heated at 70 °C in a water bath and stirred at 150 rpm for 1 h. The solution was adjusted to pH 7–7.5. Then, a rotary evaporator was used at 50 °C and 50 mbar for 1 h, and the solution of liquid resole monomer was removed, collected and stored at −4 °C for further use.

#### Processing

The process started with the preparation of a suspension yield with the diluted chitosan and resole solution inside a syringe, which was then ejected into glass cylinders (2 cm base × 1.6 cm height) as molds. These recipients were cooled down to −70 °C and then carried into a laboratory and freeze-dried (Freeze-Dryer system Ilshin Biobase TFD8501, Komachine, Seoul, Republic of Korea), using a vacuum at 35 mTorr for 24 h. Without removing the glass recipient after freezing, the integrity of the matrix attached to the glass cylinder area was ensured. The last step was a thermal treatment at 60 °C for 24 h. Formulation of different samples is shown in Table 5.

### 4.3. CS-x Aerogel Characterization

The morphology of the chitosan/resole CS-x aerogels was characterized by Scanning Electron Microscopy (SEM) (HITACHI TM-1000, Tokyo, Japan) to observe microstructure morphology and internal porous structure. Functional groups of the solid aerogel were determined by a Fourier-Transform Infrared Spectrometer with Attenuated Total Reflection (Bruker Alpha II, Billerica, MA, USA).

Surface composition was analyzed with X-ray Photoelectron Spectroscopy with a monochromatic Al Kα X-ray source (hν = 1486.7 eV) (Phoibos 150, SPECS, Berlin, Germany) operated at 250 W. Survey spectra data were collected using a step size of 0.5 eV and a pass energy of 40 eV. High-resolution spectra of core levels C1s, O1s, N1s and Cr2p were collected using a step size of 0.1 eV and a pass energy of 15 eV. The corresponding peak areas were calculated using software AAnalyzer 2.23 [42] to calculate concentration. Deconvoluted spectra for all core levels were presented using simultaneous fitting and the corresponding chemical shift reference position at 285.4 eV for the main C1s peak on CS-x aerogels and 284.8 eV for resole [41]. To compensate for the charge of non-conducting samples, an electron flood gun for charge neutralization was operated during measurements. The cross-section and sensitive factor values were taken from theoretical calculations [67,68].

### 4.4. Swelling Experiments

Also, a test of the degree of swelling (S%) and porosity was calculated to identify the water absorbed by the matrix; all the experiments were performed at 25 °C. This was calculated by weighing the CS/R-x aerogels. The initial mass was weighed before absorption, $W_{d}$ (dry state); then, the CS/R-x aerogels were immersed in distilled water until they were saturated and then weighed again, $W_{W}$ (wet state). Excess was removed by allowing drips to fall by gravity for 5 s. The *S*% was calculated with the following Equation (1) [19,39]:

(1) $$S\%=\frac{W_{W}-W_{d}}{W_{d}}$$

where *W_w_* (g) and *W_d_* (g) are the mass of the sample in the wet and dry states. The apparent density indicates the quantity of hollow spaces measured from volume or weight, which directs the influence on adsorptive capacity; it was measured with the gain of volume solution captured in the pores of CS/R-x aerogels with the following Equation (2). Also, the porosity can be measured with this criterion, Equation (3) [50].

(2) $$aparent\;density=\frac{W_{w}-W_{d}}{\rho_{w}V_{c}}$$

where $\rho_{W}$ is the density (g·cm^−3^) of water retained in the sample and the cylinder volume $V_{c}$ is 4.02 cm^3^.

(3) $$P=\frac{V_{e}}{V_{t}}*100$$

where $V_{t}$ (cm^3^) represents the total volume, $V_{e}$ (cm^3^) represents the volume of the empty spaces, and *P* represents the porosity. Experiments were made in triplicate, and the data presented were mean values.

### 4.5. Shore A Hardness Measurements, Mechanical Characterization

Shore durometer measurements were performed, using Shore A for thermoplastic elastomers. Measures were made according to ASTM D2240 [69], Ref. [40]. Assessments were taken in a parallel direction to the pore orientation (Indenter stroke 0–2.5 mmm, end pressure 0.55–8.06 N, were performed according to ISO 7619-1:2004, which is identical to GB/T 531.1-2008. standard GB/T 531.1-2008/ISO7619-1:2004) [70].

### 4.6. Chromium Solutions

Stock solution of Cr(VI) was prepared by dissolving 2.832 g of K_2_Cr_2_O_7_ (grade ACS) in 1L of distilled water. A calibration curve was in the range of 0.1 to 1.0 mg g^−1^ made from a 1000 mg·g^−1^ standard solution (Golden Bell, Materiales y Abastos Especializados, Zapopan, México).

#### 4.6.1. Batch Adsorption Experiments

Batch adsorption experiments were conducted at 25 °C, cylindrical pieces of the different CS/R-x aerogels were placed with 10 mL of concentration solution of 100 ppm of Cr(VI) in batches for 24 h, and CS/R-x aerogels were immersed with original pH (5–5.5) and static conditions. The remaining concentration was diluted into the calibration curve range, adjusting pH with a few drops of HNO_3_ and adding 2 mL of 1,5-diphenylcarbazide (previously diluted 0.0250 g in 50 mL acetone) to each sample for complexation. The Cr(VI) concentration was determined colorimetrically at 540 nm by a UV-Vis spectrophotometer (UV-5600, Metash Instruments, Shanghai, China). The complexation procedure was made according to APHA-AWWA-WEF (2012) and NMX-AA-044-SCFI-2014 [8,39], and uptake of Cr(VI) was calculated according to Equations (4) and (5):

(4) $$Q_{e}=\frac{C_{o}-C_{e}}{m}$$

(5) $$\mathrm{percentage}\;\mathrm{removal}=\left(\frac{C_{o}-C_{t}}{C_{o}}\right)*100$$

where $Q_{e}$ represents the amount of Cr(VI) adsorbed per gram of CS/R-x aerogel (mg·g^−1^), m is the mass of adsorbent (mg·L^−1^), $C_{o}$ is the initial concentration and $C_{e}$ is the concentration at equilibrium, and $C_{t}$ is the final concentration at the end time of the adsorption process (mg·L^−1^). Experiments were made in triplicate with and without thermal treatment for reticulation; the data presented were mean values.

#### 4.6.2. Column Adsorption Experiment

**Figure 8:**
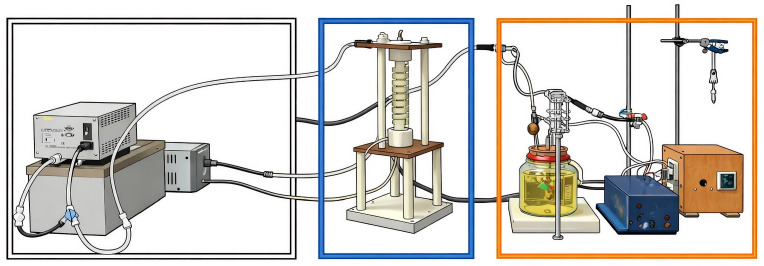
Schematic representation of the experimental continuous adsorption setup, showing the temperature recirculation unit (white box), the differential fixed-bed adsorption column packed with stacked CS/R aerogel tempered glass tubes (blue box), the mechanically agitated feed tank with thermal jacket (orange box), which was operating under closed-loop recirculation with a peristaltic pump for flow control. A scheme was created using ChatGPT 5.3 Go, based on real photographs of the experimental setup.

Column adsorption experiments (Figure 8) were conducted using a differential fixed-bed configuration, specifically designed to evaluate the dynamic performance of QS-x porous aerogel material, which has two phases (solid–liquid), and to promote homogeneous dispersion of the contaminant under continuous operation. The adsorption column consisted of a transparent tempered glass tube fitted with nylon plugs and internal couplings to ensure axial flow. The column had an internal diameter of approximately 2 cm and an effective bed length of 22 cm of packed material of approx. 0.8 g. The adsorbent bed was formed by stacked cylindrical CS/R aerogel tubes, arranged to operate under differential conditions and minimize axial dispersion [71].

The feed solution was stored in a double-walled, stirred glass tank equipped with inlet and outlet ports and a shaft-driven mechanical stirrer with 7 and 14 min intervals to maintain a constant concentration throughout the experiment. Solution circulation was achieved using a peristaltic pump (Masterflex^®^ 7518-00, Barrington, IL, USA) with an Easy-Load L/S pump head), which operated at a flow rate of approximately 90 to 100 mL min^−1^ and ensured stable hydrodynamic conditions during operation. The system was operated in closed-loop recirculation mode at ambient temperature (25 °C) without pH adjustment. The adsorption column effluent was collected from the stirred tank using a syringe to determine adsorption kinetics. Before each kinetic analysis, a conditioning treatment was carried out for 4 h until a neutral pH was detected in the adsorbent column. This pH was measured by taking 2 mL aliquots at different contact times. The kinetics were performed with adsorption tests at different Cr(VI) concentrations, ranging from 20 to 140 g·mg^−1^, over a maximum time of 24 h, at which point the concentrations reached equilibrium. The Cr(VI) removal efficiency was calculated from these data details of column assembly in Supplementary Materials.

The adsorption kinetics of the Cr(VI) was studied on the CS85/R15 aerogels, using pseudo-first order and pseudo-second-order kinetic models, Equations (6) and (7), respectively [72].

(6) $$ln{(q}_{e}-q_{t})=lnq_{e}-K_{1}t\;\;$$

(7) $$\frac{t}{q_{t}}=\frac{1}{k_{2}\;q_{e}^{2}}+\frac{t}{q_{e}}\;$$

where *q_e_* and *q_t_* (g·mg^−1^) indicate the adsorption capacities at equilibrium time *t* (h), *k*_1_ (min^−1^) is the constant rate for the pseudo-first order, and *k*_2_ (g·mg^−1^·min^−1^) is the constant rate of the pseudo-second order.

Following the isotherm analysis, we use the Langmuir model for fitting, and adsorption takes place at specific, equivalent surface sites until a monolayer is completed, with no interaction between the adsorbed molecules; under these assumptions, the equilibrium can be described by the classical Langmuir Equation (8).

(8) $$Q_{e}=\frac{Q_{m}K_{L}C_{e}}{1+K_{L}C_{e}}\;$$

The Freundlich isotherm [73] accounts for heterogeneous surface energies and multilayer adsorption, permitting interactions between adsorbate molecules and the surface, a behavior commonly linked to chemisorption, as described by the Freundlich Equation (9).

(9) Qe=KFCe1n  

where *Q_e_* (mg·g^−1^) indicates the adsorption capacity at equilibrium in the solid phase, *Q_m_* (mg·g^−1^) represents the maximum quantity of adsorbed molecules on to the surface, *C_e_* (mg·L^−1^) represents the concentration of the molecules at equilibrium, *K_L_* (L·mg^−1^) represents the Langmuir constant, and *K_F_* (mg·g^−1^) and 1/n are Freundlich constant being an indicator of heterogeneity and intensity [31,74].

## Figures and Tables

**Table 1:** Swelling percentage (S%).

Short Name	Average Dry Weight After Curing (g)	Average Wet Weight (g)	Swelling Percentage S%	Apparent Density (g cm^−3^)
CS100/R0 ^1^	0.103 +/− 0.005	5.340 +/− 0.425	5068	1.305
CS95/R05 ^1^	0.146 +/− 0.020	3.376 +/− 0.172	2197	0.805
CS90/R10 ^1^	0.166 +/− 0.015	2.893 +/− 0.090	1633	0.680
CS85/R15 ^1^	0.193 +/− 0.015	2.183 +/− 0.141	1029	0.496
CS80/R20 ^1^	0.200 +/− 0.075	1.756 +/− 0.025	778	0.388
CS75/R25 ^1^	0.236 +/− 0.005	1.586 +/− 0.142	570	0.336
CS70/R30 ^1^	0.480 +/− 0.026	1.553 +/− 0.112	224	0.268
CS65/R35 ^1^	0.483 +/− 0.005	1.460 +/− 0.098	202	0.243
CS60/R40 ^1^	0.573 +/− 0.020	1.513 +/− 0.112	164	0.234
CS55/R45 ^1^	0.560 +/− 0.047	1.376 +/− 0.040	146	0.204
CS50/R50 ^1^	0.670 +/− 0.045	1.320 +/− 0.121	97	0.162

^1^ combination with respect to a volume of 4 mL.

**Table 2:** Elementary composition from XPS analysis by component deconvoluted and its corresponding defined binding energy (eV) (with respect to main elements except hydrogen %).

Sample	Be (eV)	C1s	Be (eV)	O1s	Be (eV)	N1s
Resole	284.8	68%	533.0	18%		
	286.1	14%	539.9	Shake off		
	291.5	Shake off				
CS75R25	285.3	31%	534.3	26%	401.3	2.3%
	286.8	21.3%	533.5	0.5%	399.9	0.1%
	287.6	15%				
	288.8	4%				
	292.4	Shake off				
CS85/R15	285.4	22%	534.3	26.2%	401.3	5.4%
	286.9	23.3%	533.0	2.4%	399.9	0.6%
	287.7	12%	532.0	1.2%		
	289.9	4%				
	283.9	2%				
CS95/R05	285.4	16%	534.7	15%	401.3	4.8%
	286.8	29%	533.4	13%	399.9	1.5%
	288.2	16%	532.1	2%		
	289.5	2.4%				
	284.5	3%				

**Table 3:** Isotherm analysis.

Freundlich				Langmuir		
log K_f_ (L/g)	K_f_	1/*n*	R^2^	Qmax (mg·g^−1^)	b (L/mg)	R^2^
0.7248	2.507	0.6667	0.9786	100.8	0.806	0.9634

**Table 4:** CS-based adsorbent comparison.

Adsorbent	pH	Qmax (mg·g^−1^)	Temperature (°C)	Contact Time (h)	Adsorbent Dose (g/L)	Kinetic Model	Isotherm
CS flakes [8]	3	22.09	25	4	13	PSO	Langmuir
Crosslinked CS resin [47]	2	45.87	40	1	5	PSO	Langmuir/ Temkin
Electrospun CS nanofibers [32]	3	131.58	25	8	10	PSO	Freundlich/ Langmuir
CS/cobalt ferrite nanofibrous [63]	2–3	175.2–178.2–179.1	25–35–45	1	5	PSO	Freundlich/ Langmuir
Nylon-supported adsorptive membrane [28]	4	10.794	25	1	NR	PSO	Langmuir
Covalent organic frameworks cellulose aerogel [64]	3	411.12–349.66–307.73	15–25–35	12	2.5	PSO	Langmuir
PDA-modified CS aerogel beads [65]	2	374.4	NR	24	NR	PSO	Langmuir
CS reinforced with hard multi-walled carbon nanotube aerogel [21]	3	367.6	30	2	2.5	PSO	Langmuir
CS85/R15 this work	5–5.5	100.8	25	24	0.8	PSO	Freundlich/ Langmuir

**Table 5:** Formulation of CS/R aerogel adsorbents with mass balance based on CS solution.

Short Name	Average Dry Weight After Curing (g)	CS Diluted on Weight (g)	CS Percentage on Weight CSW%
CS100/R0 ^1^	0.103 +/− 0.005	0.079	76.69%
CS95/R05 ^1^	0.159 +/− 0.020	0.075	47.16%
CS90/R10 ^1^	0.199 +/− 0.015	0.071	35.76%
CS85/R15 ^1^	0.227 +/− 0.015	0.067	29.51%
CS80/R20 ^1^	0.282 +/− 0.075	0.063	22.34%
CS75/R25 ^1^	0.247 +/− 0.005	0.059	23.88%
CS70/R30 ^1^	0.290 +/− 0.026	0.055	18.96%
CS65/R35 ^1^	0.315 +/− 0.005	0.051	16.19%
CS60/R40 ^1^	0.333 +/− 0.020	0.047	14.11%
CS55/R45 ^1^	0.338 +/− 0.047	0.043	12.72%
CS50/R50 ^1^	0.406 +/− 0.045	0.039	9.60%

^1^ Combination with respect to a volume of 4 mL, ρCS 0.987 g/mL and ρR 1.014 g/mL; (CS_X1_/_X2_): _X1_ % solution of CS with _X2_ % solution of R.

## Data Availability

Data is available from the corresponding author upon request.

## Supplementary Materials

The following supporting information can be downloaded at: https://www.mdpi.com/article/10.3390/gels12040330/s1, Figure S1: Adsorption kinetics, Figures S2–S4: Isotherm models fitted to Langmuir and Freundlich and Pseudo Second Order Ho and Mckay.

## Abbreviations

CS	Chitosan
CSW%	Chitosan percentage on weight %
R	Resole solution (prepolymer)
RW%	Resole percentage on weight %
