# Supplementary material for: Freeze-Cast Chitosan/Resole Aerogels: Effect of Resole Fraction on Properties and Their Efficiency for Cr(VI) Uptake
Source: Gels. 2026 Apr 15;12(4):330. doi: 10.3390/gels12040330 (PMC13115801; doi:10.3390/gels12040330)
Supplement: Supplementary file 1 [file gels-12-00330-s001.zip › gels-4241803-supplementary.pdf]

Supplementary information

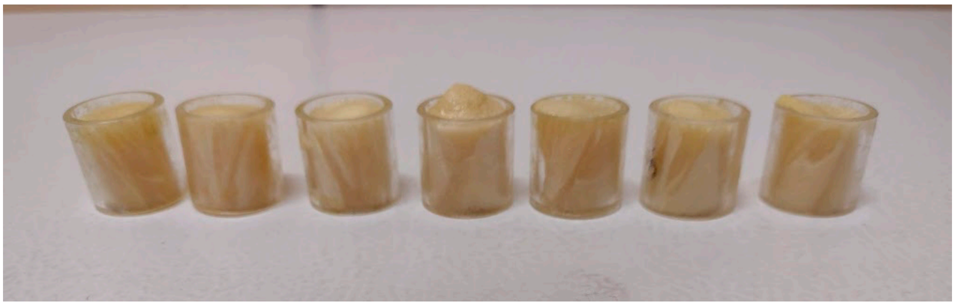

Photograph of glass tubes for differential bed in CS-x column processed aerogels

Column System Assembly and Operation

The experimental system was configured in stages and divided into three functional blocks, following the approach described by Martinez-Madrigal (2020).

Block 1 Fixed-bed adsorption column

This block consists of a 22 cm-long fixed-bed column, assembled from seven stacked tubular segments (2.0 cm diameter × 1.6 cm thickness) packed with CS85/R15 freeze-molded aerogel monoliths. The modular tube-connection design was specifically developed for these experiments to minimize axial dispersion. The assembly includes six internal couplings (2.0 cm × 1.6 cm) and two end connectors (4.0 cm length, ½-inch internal diameter) connected to nylon-66 end caps (5.0 cm external diameter). These end caps provide fluid inlet and outlet ports for recirculation. A glass jacket (1.0 cm thickness) surrounds the column to enable temperature control and structural support for the differential-bed chamber.

Block 2 Agitated feed tank with recirculation

The feed reservoir consists of two concentric glass vessels, forming a double-walled jacketed tank. The lid contains ports for stainless-steel suction and return lines connected to a peristaltic pump, as well as a shaft-driven mechanical agitator. Agitation is applied intermittently (7–14 min intervals) to maintain homogeneous ion concentration and thermal equilibrium. An additional port allows solution sampling during operation.

Block 3.Temperature control system

A water recirculation unit maintains the system temperature at 25 °C, supplying thermal control to both the column jacket (Block 1) and the feed tank jacket (Block 2).

Prior to adsorption experiments, a wetting/conditioning protocol was applied to the packed aerogel to remove soluble residues, eliminate trapped air, promote channel percolation, and neutralize acidity. This consisted of sequential recirculation of three 1 L volumes of deionized water over 4 h, ensuring stable hydraulic and chemical conditions before kinetic measurements

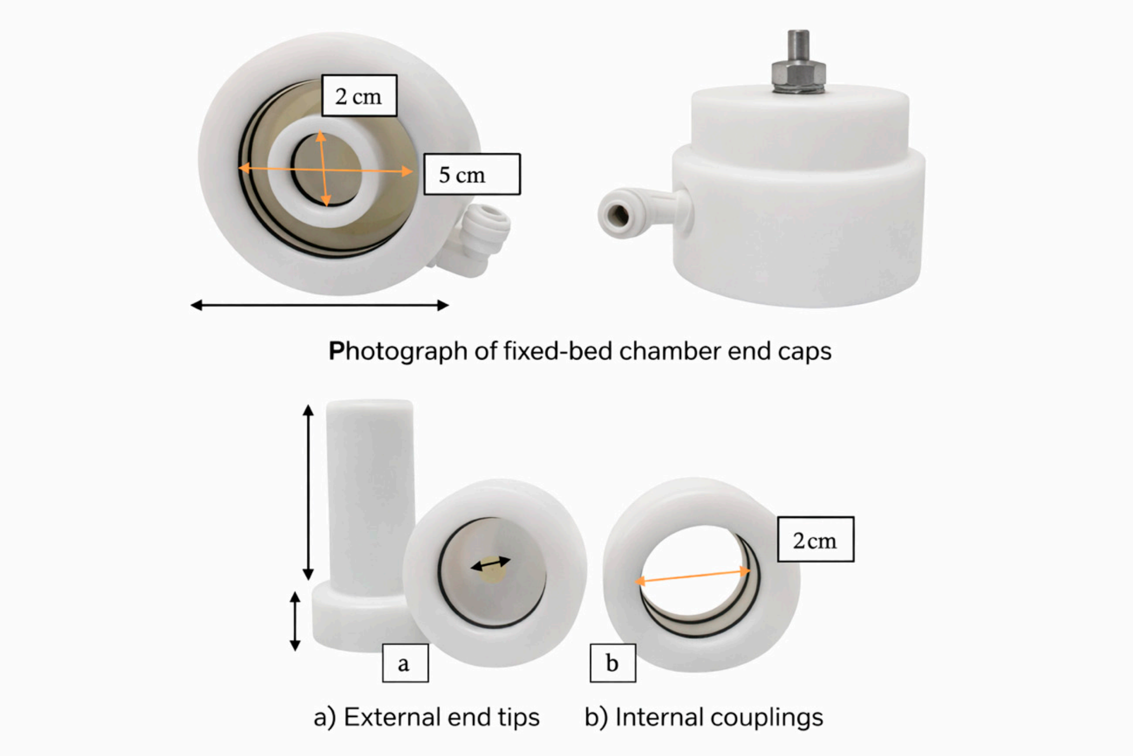

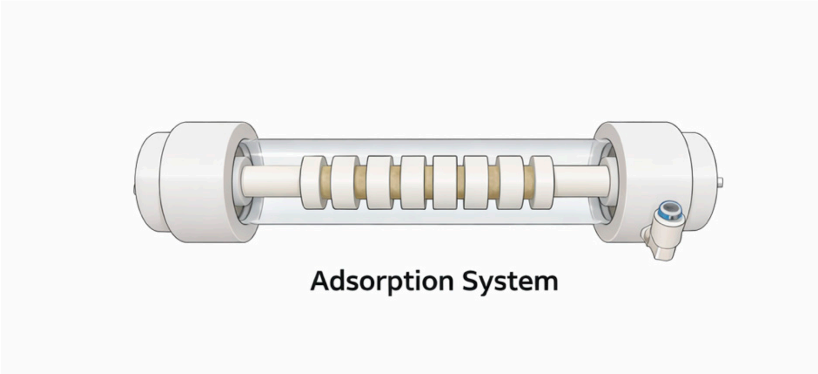

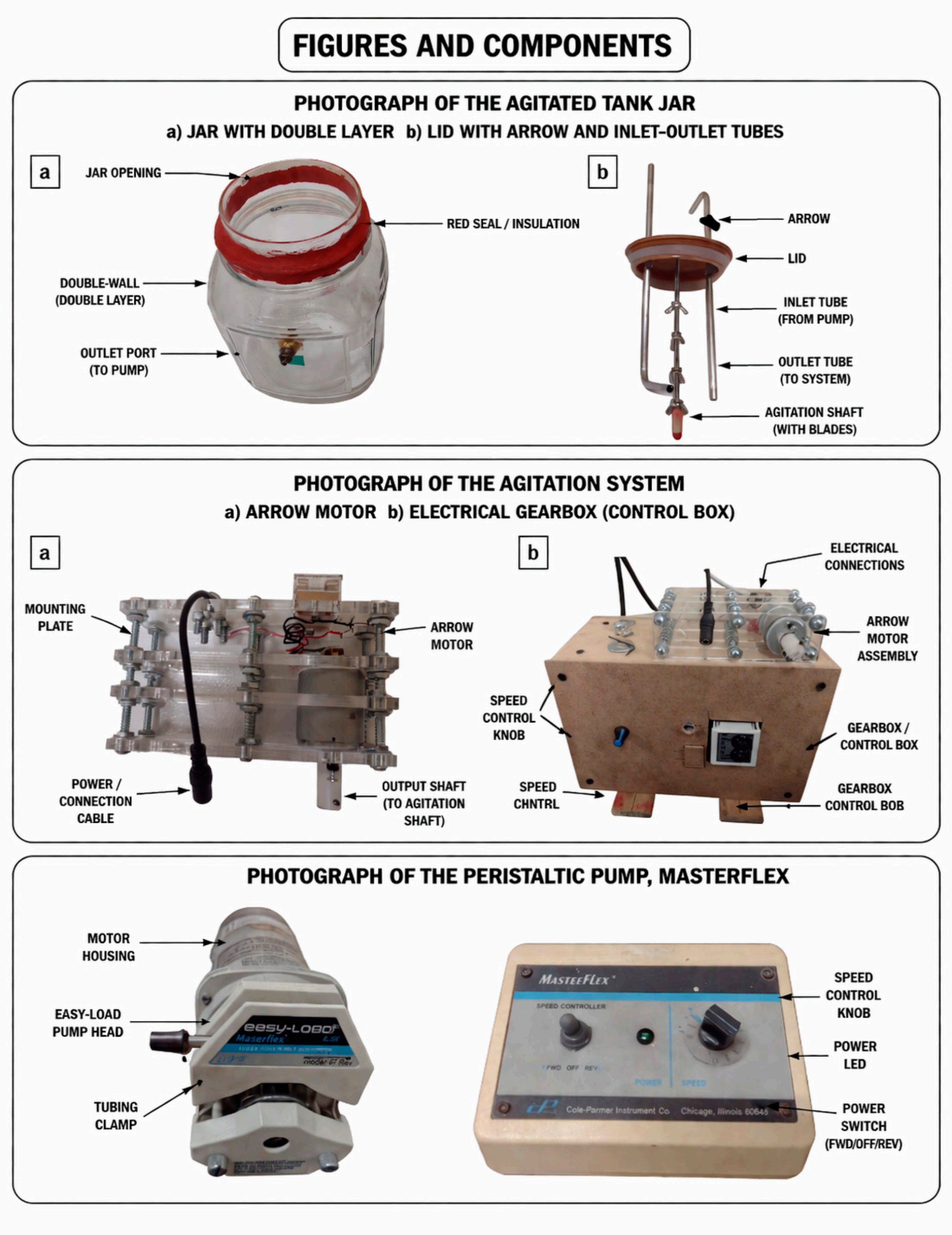

These photographs were retouched and rearranged for schematic purposes using ChatGPT 5.3 Go.

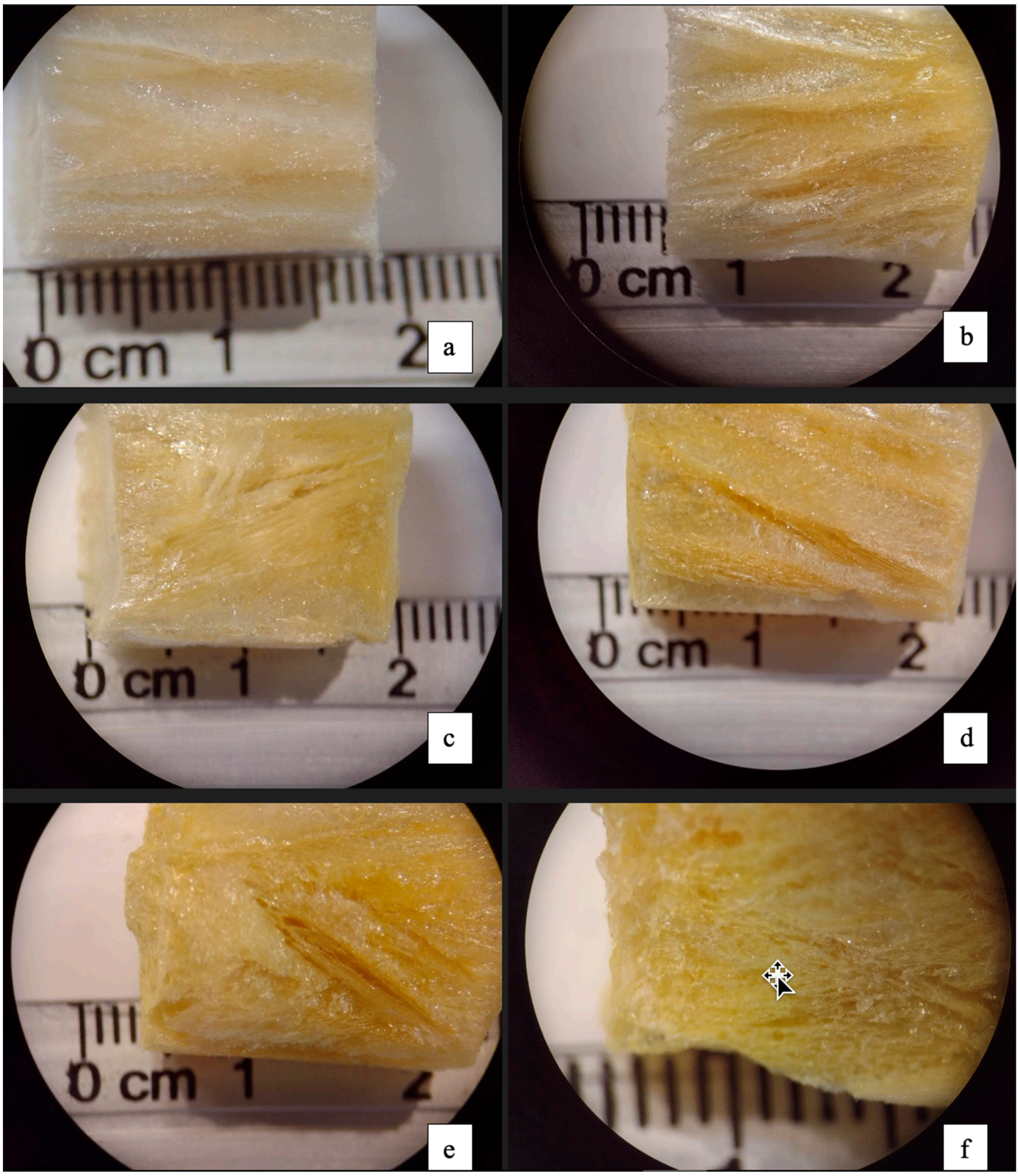

Images of samples taken cross sections in stereoscope of the monoliths with resole ratio a) QS100/0, b) QSR95/5, c) QSR90/10, d) QSR85/15, and f) QSR75/25

It can be observed how the resole rate generates the growth of ice nucleation from the glass towards the interior.

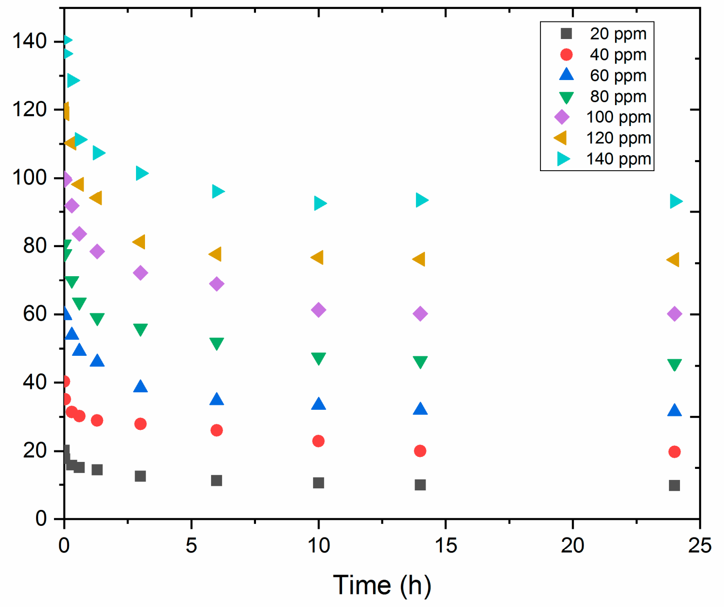

Figure S1.Adsorption kinetics

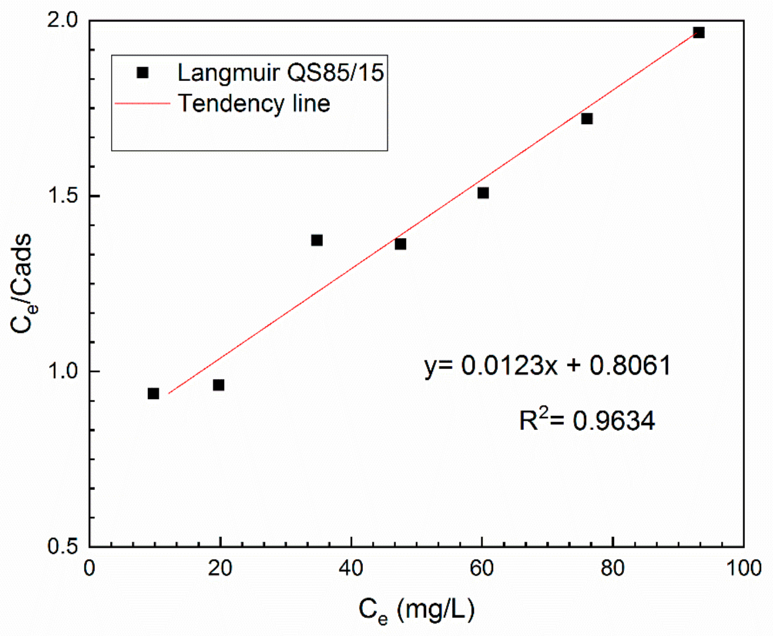

Figure S2.Isotherm model fitted to Langmuir

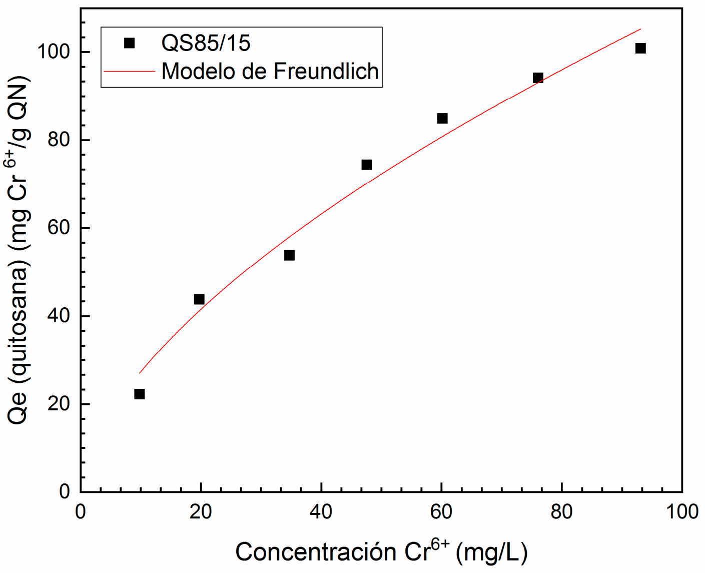

Figure S3.Isotherm model fitted to Freundlich

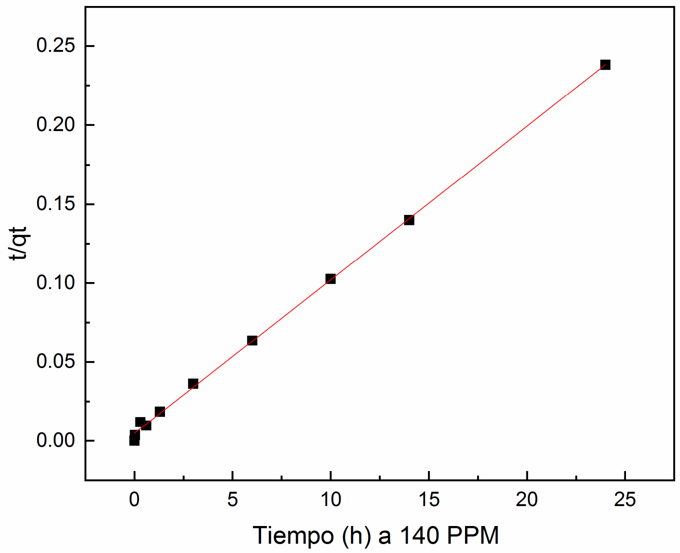

Figure S4. Kinetic data fitted to the second-order Ho and Mckay model, with an initial Cr (VI) concentration of 140 ppm
